# Rectus abdominis muscle with different abdominal pathologies: A cite to myofascial trigger point

**DOI:** 10.4274/tjod.galenos.2020.39586

**Published:** 2020-12-10

**Authors:** Fatih Bağcıer

**Affiliations:** 1 Biruni University Faculty of Medicine, Department of Physical Medicine and Rehabilitation, İstanbul, Turkey

**Keywords:** Rectus abdominis muscle, primary dysmenorrhea, myofascial pain syndrome

## Dear Editor,

I read the article, “Relation between uterine morphology and the severity of primary dysmenorrhea”^(1)^ with interest. Although the etiologic factors in primary dysmenorrhea are covered in the article, the role of muscle structures in etiology is not mentied. In this article, we would like to discuss the rectus abdominis muscle and its myofascial trigger point (MTrP), which is another underdiagnosed cause of primary dysmenorrhea and abdominal wall pain.

The rectus abdominis muscle extends from the pubic symphysis and tubercle as a beam and ends at the anterolateral aspect of the xiphoid process and superiorly in three fragments of the costal cartilages of the ribs 5-7^(2)^. The rectus abdominis muscle facilitates flexion of the trunk and stabilizes the pelvis. Contributing to the increased abdominal pressure with other abdominal muscles, it also plays a role in micturition, defecation, vomiting, and childbirth. MTrPs of the rectus abdominis muscle have been associated with pathologies such as pain in the abdominal wall and primary dysmenorrhea^(3,4)^.

Primary dysmenorrhea is a frequently encountered condition that reduces the quality of life of the patient and considerably impacts the economy of the healthcare system. Primary dysmenorrhea is a recurrent, cramping pain in the lower abdomen occurring during menstruation without any pelvic pathology^(3)^. Medical treatments, yoga, pilates and stretching exercises, massage techniques for soft tissues, and invasive approaches can be used to treat primary dysmenorrhea^(3)^. According to the literature, Gaubeca-Gilarranz et al.^(3)^ administered placebo-controlled dry needle treatment for MTrP of the rectus abdominis muscle in patients with primary dysmenorrhea. Moreover, Huang and Liu^(5)^ performed local anesthetic injection therapy to treat the MTrPs of the rectus abdominis and oblique muscles in patients with primary dysmenorrhea.

As observed by physicians, abdominal pain has been most frequently associated with intraabdominal pathologies. Therefore, numerous consultations and tests are required, and abdominal surgeries are occasionally performed, although the necessity thereof is debatable^(1)^. When patients cannot find a remedy for their pain, they visit various clinics for examinations, and certain patients are considered to have psychiatric disorders. In fact, MTrP of the rectus abdominis muscle causes abdominal wall pain. Muscolino EJ detected the MTrP of the rectus abdominis muscle in a patient with Crohn’s disease who had a complex history of medical treatments and clinical courses^(4)^. A stretching exercise program for the relevant muscle was used to treat this patient.

The diagnosis of MTrP is under-recognized in clinical practice. On examination, the possibility of MTrP localization in each muscle should be considered. MTrPs of the rectus abdominis and related muscles can have different clinical manifestations. These patients should be thoroughly evaluated and appropriate treatment modalities should be used. Therefore, randomized controlled studies with a long-term follow-up are required in this field.

## References

[1] Şentürk Ş (2020). Relation between uterine morphology and severity of primary dysmenorrhea. Turk J Obstet Gynecol.

[2] Maquirriain J, Ghisi JP, Kokalj AM (2007). Rectus abdominis muscle strains in tennis players. Br J Sports Med.

[3] Gaubeca-Gilarranz A, Fernández-de-Las-Peñas C, Medina-Torres JR, Seoane-Ruiz JM, Company-Palonés A, Cleland JA, et al (2018). Effectiveness of dry needling of rectus abdominis trigger points for the treatment of primary dysmenorrhoea: a randomised parallelgroup trial. Acupunct Med.

[4] Muscolino JE (2013). Abdominal wall trigger point case study. J Bodyw Mov Ther.

[5] Huang QM, Liu L (2014). Wet needling of myofascial trigger points in abdominal muscles for treatment of primary dysmenorrhoea. Acupunct Med.

